# Exploring the genetic variability in yield, nutritional and digestibility traits in oat grains through ruminant nutrition

**DOI:** 10.1016/j.heliyon.2024.e31541

**Published:** 2024-05-18

**Authors:** Sultan Singh, Pushpendra Koli, Shahid Ahmed, Neeraj Kumar, Maneet Rana, Rajesh Singhal, Indu, Mukesh Choudhary, Yonglin Ren

**Affiliations:** a Plant Animal Relationship Division, ICAR-Indian Grassland and Fodder Research Institute, Jhansi, UP, 284003 India; b Crop Improvement Division, ICAR-Indian Grassland and Fodder Research Institute, Jhansi, UP, 284003 India; c College of Environmental and Life Sciences, Murdoch University, 90 South Street, Murdoch, WA, 6150, Australia; d ICAR-Indian Agricultural Research Institute, New Delhi, 110012, India; e ICAR-Indian Institute of Maize Research, Ludhiana, 141001, Punjab, India; f School of Agriculture and Environment, The UWA Institute of Agriculture, The University of Western Australia, Perth, WA, 6009, Australia

**Keywords:** Avena sativa, Food energy, Forage, Genetic variability, Nutritional value, Oats grain

## Abstract

Oat is a dual-purpose crop used for both food and feed for animals. The objective of this work is to characterize oat varieties for their genetic diversity in yield, physical traits, and nutritional composition, aiming to identify potential parent varieties for breeding programs to develop new oat varieties for improved livestock feed and diverse industrial applications. To conduct, chemical analysis for protein and carbohydare fractions, energy and digestible nutrient estimated, stastical analyses performed to assess genetic variations for traits among vaieties. Significant genetic variation (*p* < 0.05) for grain yield, grain density, sieving percentage, crude protein, ether extract, neutral and acid detergent fiber, cellulose, lignin, neutral and acid detergent insoluble nitrogen were observed in grains of eight oat varieties. All protein fractions exhibited significant differences (*p* < 0.05). Total carbohydrate content ranged significantly (*p* < 0.05) from 73 % to 79 %. The grains contained higher levels of intermediately degradable starch and pectin (54.12–60.16 %) compared to the slowly degradable cell wall (26–33 %), lignin bounded cell wall (6–10 %), and rapidly degradable sugars (2–8%). Significant variation (*p* < 0.05) was observed in terms of gross energy, digestible energy, metabolizable energy, net energy for maintenance and lactation about (2 Mcal/kg dry matter), gain (1.6–1.8 Mcal/kg dry matter), total digestible nutrients, digestible dry matter, rumen degradable protein, and total digestible nutrients related to crude protein, fatty acid, neutral detergent fiber, and non-fiber carbohydrate. Organic matter and ether extract were positively associated (*p* < 0.01) with total digestible nutrients, digestible and metabolizable energy, dry matter digestible and truly digestible non fibrous cabohydrates, while neutral and acid detergent fiber and cellulose showed negative correlation. The research shows that oat varieties vary widely in their yield, physical features, and nutritional content, offering potential for breeding better varieties for both animal feed and industrial uses.

## 1 Introduction

Oats (*Avena sativa*) are an important and staple grain with diverse applications ranging from human consumption to industrial use. Oat crop is also covers large component and grown as a livestock feed globally [1]. Genus *Avena* contains up to 30 recognized species with ploidy levels of diploid, tetraploid and hexaploid [2]. During 2022, global oat grain production was 22.7 million metric tons, Russia was the second global oat producer, after the European Union, with an oat production amounting to about 4.1 million metric tons [3]. Nearly 74 % of total oat grain production goes to livestock feed [4]. The potential of oat grains to replace other cereal grains and economise dairy production has spurred the development of new oat varieties with superior nutritional quality [5]. In India, oats are an important energy supplement for small ruminants reared on pasture. Studies on its nutritional and physical characteristics have been carried in the context of industrial use and human nutrition [6]. Physical traits are primarily governed by the genotype and environment interaction, and hence exhibit differences in milling properties. The energy of a feed is utilized with varied efficiencies depending on the source, intake and animal function (maintenance, milk, growth). Additionally the microbial composition also have influence on feed efficiency in cattles [7]. In ruminant diets, grains high in starch and low in acid detergent fiber or the insoluble fraction are preferred as such composition combined with high particle size on dry rolling is associated with a higher growth rate and slower digestion rate [8,9]. As cereal grains differ in rumen degradation rates, their feeding value on supplementation to roughage-based diet may also differ. Thus, precise estimation of protein, energy, and nutrient digestibility is essential for balanced/improved diet formulation. The use of oat grains as a component of animal feed not only enhance milk production it also help in reduction of methane emissions in cattle [10,11].

Given oat's importance as a forage crop and as animal feed, the recent analysis of eight oat varieties for their nutritional and genetic variability has shed light on the unique attributes of each, presenting both desirable and undesirable traits. Despite extensive research on oat grains, there appears to be a limited body of work specifically addressing the correlation between genetic variability and nutritional content [12]. Therefore, a comprehensive understanding of the genetic mechanisms governing these traits remains elusive. Based on the yield, nutritional and digestibility profile of oat varieties from this study provides a choice to farmers for cultivation of suitable varieties. Moving forward, pinpointing specific genetic markers linked to these traits could revolutionize oat breeding programs. Furthermore, diverse parents can be used to develop mapping population and investigate the genetics of yield and quality traits [13]. Given the understanding above and recognizing a research gap regarding the absence of an appropriate variety of oat grains that comprehensively addresses both nutritional and animal health aspects, this study aims to analyze different types of oats to understand how they vary genetically in terms of how much they produce, their physical characteristics, and what nutrients they contain. By doing this, we hope to find oats that could be good parents for making new types of oats that are better for feeding animals and for different uses in industry. This will help promote oats as a healthy choice for feeding animals and provide guidance on how to grow them best in different environments to get the most out of them.

## 2 Materials and methods

### 2.1 Location, experimental layout and sample processing

The experiment was carried out at the Central Research Farm of ICAR-Indian Grassland and Fodder Research Institute, Jhansi, India (25.5114° N, 78.5337° E) at 271 m altitude. The soil in the area was inceptisol having a loamy and clay loam surface texture. The average minimum and maximum temperature during cropping season (November to mid-April) ranging from 6.3 °C to 17.0 °C and from 22.6 °C to 37.5 °C, respectively. The relative humidity of the experimental period varied between 59 and 73 %. For field preparation, one ploughing with mould board plough followed by two cross harrowing and planking was done. Crop was grown under irrigated conditions where pre sowing irrigation was given for proper germination followed by five irrigations during different stages of crop maturity. One manual weeding was done after 30 days of crop growth. Eight oat varieties viz. JHO2009-1, JHO-851, JHO2012-2, JHO99-2, JHO2010-1, JHO2000-4, JHO-822 and JHO99-1 were grown (60 kg N and 40 kg P_2_O_5_/ha was applied before sowing) in 3 m row plots with three replications in November 2018–19. At day 45 after sowing, a top dressing of 20 kg N/ha was applied. Plants were spaced 25 cm between rows and distributed according to a randomized complete block design. Grain samples were collected and dried at 100 °C for 72 h for dry matter (DM) estimation, and at 60 °C for 72 h for chemical/biochemical estimations. Dried samples were ground through a 1 mm sieve using a Willey mill and then stored for further nutritional and *in vitro* analyses.

### 2.2 Grain yield and physical characters of oat grain

Grain yield and physical traits (hull content, groat content, grain density and sieving percentage) were recorded. Grain density was measured by dividing the weight of a quantity of seeds by its volume. Sieving percentage was expressed as grain retained in a 2 mm slotted screen after 2 min of mechanical shaking. The screened samples were de-hulled mechanically for calculation of hulling and groat percentage. For milling/dough recovery, 100 g grain samples were ground in a 500 W grinding machine (Wiley mill) for either 30 s or 60 s followed by sieving through an 850-μm mesh.

### 2.3 Chemical analyses

Oat grain samples were analyzed for DM, crude protein, ether extract and ash following the Association of Official Analytical Chemists guidelines [14]. Neutral detergent fiber (NDF) and acid detergent fiber (ADF) were determined [15] using a Pelican Fiber apparatus (Fibre tech, Fibra Plus FES 6, Pelican India). Lignin was determined by solubilizing cellulose with sulfuric acid in the ADF residue [16]. Hemicellulose was calculated as the difference between NDF and ADF, and cellulose was estimated as ADF minus lignin in the sequential analysis.

### 2.4 Crude protein fractionation

Grain crude protein (CP) fractions were partitioned into five fractions as per Cornell Net Carbohydrate and Protein System (CNCPS) [17] as modified by Licitra et al. [18]. These were.

i. P_A_, non-protein N, estimated as the difference between total N and true N precipitated with sodium tungstate (0.30 M) and 0.5 M H_2_SO_4_,
ii. P_B1_, buffer soluble protein, calculated as the difference between true protein and buffer-insoluble protein, estimated with borate phosphate buffer (pH 6.7–6.8) and freshly-prepared 0.10 sodium azide solution.
iii. P_B2_, neutral detergent soluble protein, was estimated as buffer-insoluble protein minus ND insoluble protein.
iv. P_B3_, acid detergent soluble protein, was estimated as the difference between ND insoluble protein and acid detergent insoluble CP.
v. P_C_, indigestible or bound to lignin.

### 2.5 Carbohydrate fractionation

Carbohydrate fractions of oat grains were estimated following CNCPS [17]. It classifies carbohydrate fractions according to degradation rate into four fractions, as follows.

i. C_A_ rapidly degradable carbohydrates, including sugars;
ii. C_B1_ intermediately degradable starch and pectins;
iii. C_B2_ slowly degradable plant cell wall, and
iv. C_C_ unavailable/lignin-bound plant cell wall.

Total carbohydrates (g/kg DM) were calculated by subtracting CP, EE and ash contents from 1000. Structural carbohydrates (SC) were calculated as NDF-NDIP, and non-fiber carbohydrates were estimated as tCHO-SC [19]. For starch estimation, samples were extracted with ethanol to solubilize free sugars, lipids, pigments and waxes. The residue rich in starch was solubilized with perchloric acid and the extract was treated with anthrone–sulfuric acid to determine glucose colorimetrically using standard glucose [20].

### 2.6 Energy and digestible nutrients

Grain digestible dry matter (DDM), total digestible nutrients (TDN) and net energy (NE) for different animal functions i.e., lactation (NE_L_), growth (NE_G_) and maintenance (NE_M_) were calculated using equations of Undersander et al. [21]. Digestible energy (DE) and net energy (NE) were calculated using the equations of Fonnesbeck et al. [22] and Khalil et al. [23], respectively. Gross energy (GE), truly digestible non fibrous carbohydrates (tdNFC) and truly digestible crude protein (tdCP), truly digestible neutral detergent fiber (tdNDF) and truly digestible fat (tdFA) and rumen degradable protein (RDP) were calculated as:

(1) GE = 4299 + 7 × CP+ 53 × EE [24]

(2) tdNFC = 0.98 × (100- (NDF−NDIP) + CP + EE + Ash)) × 1 [25]

(3) tdCP = CP × Exp (−1.2 × (ADIP/CP) [25]

(4) tdFA = EE−1

(5) tdNDF = 0.75 × ((NDF-NDICP)-ADL) × (1−(ADL/NDF−NDICP))^0.667^)

(6) RDP= (P_B1_ × 2.6)/(2.6 + 0.02) + (P_B2_ × 0.096/0.095 + 0.02) + (P_B3_ × 0.005/0.005 + 0.02)

(7) Y_ijk_ = μ+s_i_+ε_ijk_

### 2.7 Statistical analysis

Analysis of variance was carried out in SPSS v20.0 using eq. (7) where Y_ij_ individual observation, μ = population mean, s_i_ = variety effect (i = 1−8) and εij is residual error for estimation of genetic variation among the varieties for grain yield, physical traits and nutritional traits. Duncan's multiple range test was applied to determine significant differences among oat varieties (at *p* < 0.05) for grain yield, physical traits, chemical composition, carbohydrate and protein fractions, energy values and digestible nutrients. Principal component analysis and factor map was plotted using ‘FactoMineR’ package in R program to understand the relationship between different milling and yield component traits. Clustered correlation for grain yield and physical traits along with chemical constituents and nutritional traits among eight oat genotypes were plotted using ‘ggplot2’ package in R program.

## 3 Results

### 3.1 Grain yield, physical and milling traits

Grain yield and 1000 grain weight of oat varieties varied (*p* < 0.05) from 1.25 to 2.07 t/ha and 16.17–52.57g, respectively (Table 1). JHO-2009-1 exhibited highest grain yield (2.07 t/ha) followed by JHO-2012-2 and JHO-2010-1, whereas JHO-851 and JHO-99-1 were poor grain yielders. Grain density (kg/hl) and density percentage varied (*p* < 0.05) amongst varieties and being highest for JHO-822 (268 and 53.60) and lowest for variety JHO-851 (211 and 42.20). Sieving % was maximum for JHO-99-2 (92.67) and minimum for JHO-851 (19.0). Grain length and width ranged (*p* < 0.05) between 1.17-1.50 cm and 0.30–0.40 cm, respectively. Flour recovery at 30 and 60 s times differed (*p* < 0.05) across varieties, while hull and groat contents were comparable at 30.97*-*35.22 % and 64.77*-*69.02 %, respectively.

**Table 1:** Grain yield and physical characteristics of eight oat varieties.

Parameters	JHO-822	JHO-851	JHO-99-1	JHO-99-2	JHO-2000-4	JHO-2010-1	JHO-2009-1	JHO-2012-2	Mean	SEM	*p*-value
Grain yield (t/ha)	1.42^bc^	1.26^a^	1.25^a^	1.32^ab^	1.53^c^	1.82^d^	2.07^e^	1.83^d^	1.56	0.061	0.0001
Grain wt_1000_ (g)	42.63^c^	16.17^a^	41.60^c^	52.57^e^	36.97^b^	52.43^e^	40.40^c^	48.13^d^	41.36	2.284	0.0001
Grain density (kg/hl)	268.00^g^	211.00^a^	236.00^c^	241.00^d^	252.00^e^	242.00^d^	258.67^f^	230.00^b^	242.33	3.457	0.0001
Grain density %	53.60^g^	42.20^a^	47.20^c^	48.20^d^	50.40^e^	48.40^d^	51.73^f^	46.00^b^	48.47	0.691	0.0001
Sieving %	66.53^b^	19.00^a^	73.93^c^	92.67^f^	81.33^d^	84.6^e^	72.73^c^	80.20^d^	71.38	4.41	0.0001
Hull content %	32.45	34.39	34.64	35.22	33.84	30.97	32.2	32.12	33.228	0.529	0.717
Groat content %	67.54	65.60	65.35	64.77	66.15	69.02	67.79	67.87	66.618	0.529	0.717
Dough recovery _100 (g)_ 30sec	31.03^b^	26.00^a^	33.33^c^	26.00^a^	31.00^b^	30.83^b^	26.00^a^	25.37^a^	28.69	0.649	0.0001
Dough recovery _100 (g)_ 60sec	68.03^g^	59.13^c^	60.37^d^	63.03^e^	57.10^a^	64.03^f^	61.07^d^	58.07^b^	61.35	0.702	0.0001
Seed length (cm)	1.37^c^	1.17^a^	1.40^d^	1.50^e^	1.37^c^	1.33^b^	1.40^d^	1.40^d^	1.367	0.033	0.0001
Seed width (cm)	0.40^c^	0.30^a^	0.40^c^	0.40^c^	0.37^b^	0.37^b^	0.37^b^	0.37^b^	0.372	0.011	0.0001

Means followed by different superscript letters within rows differ significantly at *p* < 0.05 level.

### 3.2 Chemical composition

Among the eight oat varieties, EE was lowest (*p* < 0.05) in JHO2012-2 (5.82) and highest in JHO2009-1 (7.78 %). Grain CP, NDF, ADF and cellulose differed (*p* < 0.05) among oat varieties. Lignin content was low in grains (2.08*-*3.11 %), but exhibited significant differences among varieties. The NDIN and ADIN in grains ranged (*p* < 0.05) between 0.266*-*0.401 and 0.056*-*0.085 % DM, respectively.

### 3.3 Protein and carbohydrate fractions

Protein fraction PA differed (P < 0.05) among varieties with lowest (5.76) in JHO2012-2 and highest (19.90 % CP) value in JHO99-1 (Table 2). Grains had higher P_B1_ (51.22*-*68.85) followed by P_B3_ (9.85–16.86) and P_B2_ (7.15*-*15.67 %CP). Lignin bound protein (*Pc*) was lowest (2.19*-*4.08 %CP) among different protein fractions. Total carbohydrates (tCHO) were lowest in JHO-851 (73.24) and highest in JHO-822 (79.27 % DM) (Table 3). Grains had higher NSC (42.02–50.37) than SC (28.02–35.70 % DM). Oat grains had highest C_B1_ (54.12–60.16) followed by C_B2_ (26.15–32.89), C_C_ (6.32–10.49) and C_A_ (2.26–7.62 % tCHO).

**Table 2:** Protein and carbohydrate fractions in grains of oat cultivars.

Parameters/cultivar	JHO-822	JHO-851	JHO99-1	JHO99-2	JHO2000-4	JHO2010-1	JHO2009-1	JHO2012-2	Mean	SEM	*p* Value
P_A_%CP	14.77^cd^	17.71^de^	19.90^e^	10.52 ^ab^	15.51^cd^	10.73 ^ab^	11.83 ^bc^	7.76^a^	13.59	0.875	0.0001
P_B1_%CP	53.02 ^ab^	59.36 ^cd^	51.22^a^	57.59^cd^	57.77 ^cd^	60.99^d^	56.42 ^bc^	68.85^e^	58.15	1.0106	0.0001
P_B2_%CP	15.38^d^	7.15^a^	10.79 ^bc^	13.23 ^cd^	11.08 ^bc^	8.53 ^ab^	15.67^d^	9.86 ^abc^	11.46	0.682	0.0001
P_B3_%CP	13.77^bc^	13.59 ^bc^	14.13 ^bc^	14.59 ^bc^	12.23 ^ab^	16.86^c^	12.72 ^ab^	9.85^a^	13.47	0.493	0.010
P_C_%CP	3.07 ^bc^	2.19^a^	3.96^d^	4.08^d^	3.40^bcd^	2.89^b^	3.35 ^bcd^	3.68 ^cd^	3.33	0.137	0.001
tCHO %DM	79.27^f^	73.24^a^	76.33 ^bcd^	77.72^de^	76.11 ^bc^	76.97 ^cde^	78.39 ^ef^	75.33^b^	76.67	0.394	0.0001
NSC%DM	49.52 ^cd^	42.06^a^	46.42 ^bc^	42.02^a^	46.45 ^bc^	47.20 ^bcd^	50.37^d^	44.37 ^ab^	46.05	0.680	0.0001
SC%DM	29.75 ^ab^	31.18^b^	29.92 ^ab^	35.70^c^	29.67 ^ab^	29.77 ^ab^	28.02^a^	30.96 ^ab^	30.62	0.518	0.001
C_A_ %tCHO	4.37^b^	4.54^b^	7.10^c^	2.26^a^	4.89^b^	3.62 ^ab^	7.62^c^	6.82^c^	5.15	0.402	0.0001
C_B1_%tCHO	60.09^c^	55.71 ^ab^	56.12 ^ab^	54.12^a^	58.56 ^bc^	60.16^c^	58.43 ^bc^	54.34^a^	57.19	0.576	0.004
C_B2_%tCHO	29.12 ^ab^	32.89 ^bc^	26.15^a^	34.01^c^	29.79 ^ab^	28.98 ^ab^	26.89^a^	31.92 ^bc^	29.96	0.664	0.005
C_C_%tCHO	6.32^a^	6.82^b^	10.49^d^	9.60^c^	6.96^b^	7.11^b^	7.09^b^	7.03^b^	7.68	0.296	0.0001

Means followed by different superscript letters within rows differ significantly at *p* < 0.05 level.

**Table 3:** Chemical composition of grains of oat cultivars (%DM).

Parameters	JHO-822	JHO-851	JHO99-1	JHO99-2	JHO2000-4	JHO2010-1	JHO2009-1	JHO2012-2	Mean	SEM	*p* Value
OM	97.17^a^	95.28^a^	96.56 ^ab^	95.42 ^ab^	96.27 ^ab^	96.11 ^ab^	96.51^ab^	95.60 ^ab^	96.11	0.200	0.230
EE	6.19^b^	6.10 ^ab^	7.42^d^	6.19^b^	7.25 ^cd^	7.09^c^	7.78^e^	5.82^a^	6.73	0.146	0.0001
CP	11.71^b^	15.93^e^	12.80^c^	11.50^b^	12.90^c^	12.06^b^	10.34^a^	14.44^d^	12.71	0.349	0.0001
NDF	30.07 ^ab^	31.58^b^	30.29 ^ab^	36.04^c^	29.99 ^ab^	30.15 ^ab^	28.29^a^	31.27 ^ab^	30.96	0.521	0.001
ADF	7.34^a^	11.33^d^	8.99 ^abc^	12.10^d^	8.78 ^abc^	9.18^bc^	7.51 ^ab^	10.60 ^cd^	9.48	0.369	0.0001
Cellulose	4.62^a^	8.10^b^	4.83^a^	8.28^b^	4.44^a^	5.67^a^	4.73^a^	7.42^b^	6.01	0.347	0.0001
Lignin	2.09^a^	2.08^a^	3.34^e^	3.11^d^	2.21 ^ab^	2.28^c^	2.32^c^	2.21 ^ab^	2.45	0.96	0.0001
Lignin %NDF	6.95^a^	6.62^a^	11.06^d^	8.63^c^	7.36 ^ab^	7.56 ^ab^	8.22^bc^	7.07^a^	7.93	0.294	0.0001
Hemicellulose	22.78 ^ab^	20.27^a^	21.29 ^ab^	23.94^b^	21.21 ^ab^	21.06^a^	20.83^a^	20.67^a^	21.50	00.350	0.116
NDIN	0.315 ^ab^	0.401^d^	0.370 ^bcd^	0.344 ^bc^	0.322 ^abc^	0.379^cd^	0.266^a^	0.312 ^ab^	0.339	0.010	0.001
ADIN	0.057^a^	0.056^a^	0.081^b^	0.075^b^	0.070 ^ab^	0.056^a^	0.056^a^	0.085^b^	0.067	0.003	0.001

Means followed by different superscript letters within rows differ significantly at *p* < 0.05 level.

### 3.4 Energy and digestible nutrients

Grains GE, DE and ME differed (*p* < 0.05) in oat varieties, with JHO-851, JHO2012-2 and JHO99-2 having lower values than JHO2009-1 and JHO-822 (Table 4). A similar trend in net energy efficiency for maintenance (NE_M_), lactation (NE_L_) and growth (NE_G_) was recorded. Grains had higher NE_M_ (2.30–2.48) than NE_L_ (20.7–2.22) and NE_G_ (1.58–1.76 Mcal/kg DM). The TDN differed (*p* < 0.05) being low in JHO 851, JHO2012-2 and JHO99-2 (89.21–91.16), and higher in JHO2009-1, JHO-822 and JHO99-1 (93.26–95.41 %). Digestible DM varied among oat varieties (79.47–83.18 %). Grains of JHO99-1 and JHO-851 had the lowest RDP (77.20–81.04), whereas JHO2012-2 and JHO2010-1 (87.64–89.05 %) had the highest RDP. The tdCP, tdFA, tdNDF and tdNFC in grains varied from 10.20 to 15.79, 4.82–6.78, 14.01–18.40 and 42.95–50.73 %, respectively.

**Table 4:** Energy, digestible dry matter, rumen degradable protein and truly digestible nutrients in grains of oat varieties.

Parameters	JHO-822	JHO-851	JHO99-1	JHO99-2	JHO2000-4	JHO2010-1	JHO2009-1	JHO2012-2	Mean	SEM	*p* Value
GE Mcal/kg	4.71^a^	4.73^b^	4.78^d^	4.71^a^	4.77 ^cd^	4.76^c^	4.78^d^	4.71^a^	4.74	0.007	0.0001
DE Mcal/kg	4.21^d^	3.98^a^	4.11^bcd^	3.93^a^	4.12 ^cd^	4.10 ^bc^	4.20 ^cd^	4.02 ^ab^	4.08	0.021	0.0001
ME Mcal/kg	3.45^d^	3.27^a^	3.38 ^bcd^	3.23^a^	3.39 ^cd^	3.37 ^bc^	3.45^d^	3.30 ^ab^	3.35	0.017	0.0001
NE_L_ Mcal/kg	2.22^d^	2.09^a^	2.16 ^bc^	2.07^a^	2.17 ^bcd^	2.16 ^bc^	2.21 ^cd^	2.11 ^ab^	2.15	0.012	0.0001
NE_M_ Mcal/kg	2.48^d^	2.33^a^	2.41 ^bc^	2.30^a^	2.42 ^cd^	2.41 ^bc^	2.47 ^cd^	2.35 ^ab^	2.39	0.014	0.0001
NE_G_ Mcal/kg	1.76^d^	1.61^a^	1.69 ^bc^	1.58^a^	1.70 ^cd^	1.69 ^bc^	1.75 ^cd^	1.63 ^ab^	1.67	0.014	0.0001
TDN%	95.41^e^	90.22^a^	93.26 ^de^	89.21^a^	93.54 ^bcd^	93.02 ^bc^	95.19^e^	91.16 ^ab^	92.62	0.481	0.0001
DDM%	83.18^d^	80.08^a^	81.89 ^bcd^	79.47^a^	82.06 ^cd^	81.75 ^bc^	83.05 ^cd^	80.64 ^ab^	81.51	0.287	0.001
RDP%	83.18^bcd^	81.04 ^ab^	77.20^a^	86.47^de^	81.91^bc^	87.64^e^	85.70 ^cde^	89.05^e^	84.02	0.854	0.0001
tdNFC%	50.15 ^cd^	43.28^a^	47.39 ^bcd^	42.95^a^	47.17^bc^	48.20 ^bcd^	50.73^d^	45.09 ^ab^	46.87	0.645	0.001
tdCP%	11.56 ^bc^	15.79^f^	12.60^d^	11.32^b^	12.73^d^	11.92^c^	10.20^a^	14.23^e^	12.54	0.348	0.0001
tdFA%	5.19^b^	5.10 ^ab^	6.42^d^	5.19^b^	6.25 ^cd^	6.09^c^	6.78^e^	4.82^a^	5.73	0.146	0.0001
tdNDF%	16.06 ^ab^	16.76 ^bc^	14.01^a^	18.40^c^	15.78 ^ab^	15.52 ^ab^	14.66 ^ab^	16.71^bc^	15.99	0.327	0.007

Means followed by different superscript letters within rows differ significantly at *p* < 0.05 level.

### 3.5 Correlation of chemical constituents with energy and digestible nutrients

**Fig. 1:**
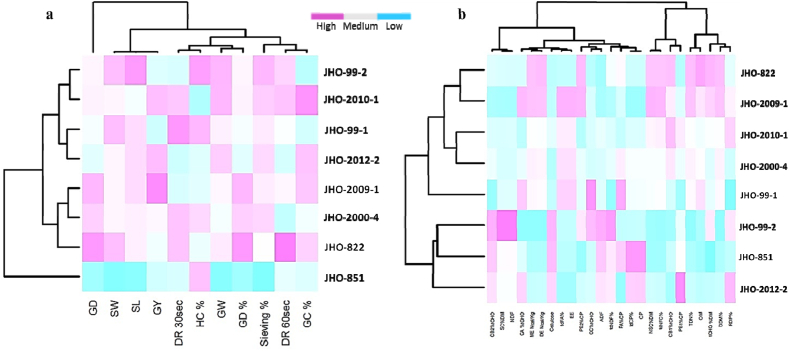
Clustered correlation matrix of (a) grain yield and physical traits, (b) chemical constituents and nutritional traits among eight oat genotypes *[GD: Grain density (kg/hl); SW: Seed width (cm); SL: Seed length (cm); GY: Grain yield (t/ha); GW: Grain wt*_*1000*_*(g); DR* 30sec*: Dough recovery*_*100 (g)*_ 30sec*; DR* 60sec*: Dough recovery*_*100 (g)*_ 60sec*; Sieving %; GC: Groat content %; HC: Hull content%]*.

Grain OM and EE had a positive correlation (*p* < 0.01) with TDN, DE, ME, DDM and tdNFC (Table 5a), and a negative correlation (*p* < 0.01) with tdNDF. Based on significant correlation values for chemical constituents and energy traits, varieties were clustered into two broad groups. The first group consisted of varieties JHO-851, JHO 2012-2, JHO-99-2, and the second group was of JHO-99-1, JHO-2000-4, JHO 2010-1, JHO-2009-1, JHO-822 (Fig. 1b). Grain CP, NDF, ADF and cellulose were negatively correlated (*p* < 0.01) with TDN, DE, ME, DDM, tdNFC and tdFA. The protein P_A_ fraction was also negatively associated (*p* < 0.01) with RDP and tdNDF, while P_B1_ was positively (*p* < 0.01) correlated with RDP and tdCP and negatively correlated (*p* < 0.01) with TDN, DE, ME, DDM and tdFA. Protein P_B2_ was negatively correlated (*p* < 0.01) with tdCP and positively (*p* < 0.01) associated with TDN, DE, ME and DDM. The NSC had a positive correlation (*p* < 0.01) with TDN, DE, ME, DDM, tdNFC and tdFA had a negative correlation (*p* < 0.01) with tdNDF and tdCP. In contrast, SC and C_B2_ had a negative correlation (*p* < 0.01) with TDN, DE, ME, DDM, tdNFC and tdFA and a positive correlation with tdNDF.

**Table 5a:** Pearson correlation of chemical constituents with energy and digestible nutrients.

	RDP	TDN	DE	ME	DDM	tdNFC	tdCP	tdFA	tdNDF
OM	−0.24	0.590∗∗	0.581∗∗	0.580∗∗	0.590∗∗	0.683∗∗	−0.343	0.244	−0.416∗
EE	−0.239	0.497∗	0.490∗	0.494∗	0.498∗	0.488∗	−0.534∗∗	1.000∗∗	−0.583∗∗
CP	−0.207	−0.483∗	−0.478∗	−0.487∗	−0.483∗	−0.564∗∗	1.000∗∗	−0.535∗∗	0.194
NDF	0.261	−0.741∗∗	−0.743∗∗	−0.735∗∗	−0.741∗∗	−0.824∗∗	0.115	−0.465∗	0.885∗∗
ADF	0.247	−1.00∗∗	−1.00∗∗	−1.00∗∗	−1.00∗∗	−0.860∗∗	0.479∗	−0.497∗	0.641∗∗
Cellulose	0.341	−0.915∗∗	−0.911∗∗	−0.915∗∗	−0.915∗∗	−0.799∗∗	0.487∗	−0.625∗∗	0.656∗∗
P_A_	−0.984∗∗	0.253	0.255	0.245	0.253	0.112	0.242	0.233	−0.427∗
P_B1_	0.728∗∗	−0.445∗	−0.443∗	−0.440∗	−0.445∗	−0.374	0.417∗	−0.445∗	0.388
P_B2_	0.029	0.415∗	0.414∗	0.419∗	0.414∗	0.387	−0.698∗∗	0.255	−0.048
tCHO	0.193	0.523∗∗	0.515∗	0.524∗∗	0.523∗∗	0.643∗∗	−0.861∗∗	0.233	−0.142
NSC	−0.09	0.864∗∗	0.861∗∗	0.860∗∗	0.864∗∗	0.997∗∗	−0.580∗∗	0.488*	−0.758∗∗
SC	0.265	−0.738∗∗	−0.739∗∗	−0.731∗∗	−0.737∗∗	−0.821∗∗	0.107	−0.464∗	0.888∗∗
C_C_%tCHO	−0.329	−0.314	−0.32	−0.31	−0.314	−0.248	−0.165	0.191	−0.086
C_B2_%tCHO	0.384	−0.718∗∗	−0.715∗∗	−0.714∗∗	−0.718∗∗	−0.870∗∗	0.387	−0.602∗∗	0.971∗∗
C_A_	−0.167	0.374∗	0.373∗	0.374∗	0.373∗	0.404∗	0.006	0.403∗	−0.658∗∗
C_B1_	−0.153	0.724∗∗	0.724∗∗	0.717∗∗	0.725∗∗	0.836∗∗	−0.359∗	0.308	−0.607∗∗

∗∗ P < 0.01.

∗ P < 0.05 level.

### 3.6 Correlation among grain yield and physical traits

Grain yield had a positive correlation with groat percentage, (r^2^ = 0.80, *p* < 0.05), while groat percentage was negatively correlated with hull content. (r^2^ = −1.0, *p* < 0.001). The 1000 grain weight had a positive correlation with seed width (r^2^ = 0.8, p < 0.05), seed length (r^2^ = 0.82, *p* < 0.05) and sieving percentage (r^2^ = 0.93, *p* < 0.001). Grain density was also correlated with seed width, dough recovery at 60 s, seed length and sieving percentage (Table 5b). Based on correlation among grain yield and milling traits, oat genotypes were placed into three broad groups (Fig. 1a).

Group 1: JHO-99-2, JHO-2010-1, JHO-99-1, JHO-2012-2,
Group 2: JHO-2009-1, JHO-2000-4, JHO-822, and
Group 3: JHO-851.

**Table 5b:** Pearson correlation of Grain milling and Physical parameters.

Trait	Grain yield	Grain wt_1000_ (g)	Grain density	Sieving	Hull	Groat	Dough recovery (_100 g_ 30sec)	Dough recovery (_100 g_ 60sec)	Seed length	Seed width
Grain yield	1									
Grain wt_1000 g_	0.357	1								
Grain density	0.333	0.457	1							
Sieving	0.342	0.929∗∗	0.527	1						
Hull	−0.801∗∗	−0.342	−0.347	−0.194	1					
Groat	0.801∗∗	0.342	0.347	0.194	−1.000∗∗	1				
Dough recovery (_100 g_ 30sec)	−0.308	0.137	0.351	0.205	−0.022	0.022	1			
Dough recovery (_100 g_ 60sec)	−0.074	0.375	0.539	0.143	−0.282	0.282	0.255	1		
Seed length	0.161	0.818∗∗	0.521	0.885∗∗	0.097	−0.097	0.030	0.179	1	
Seed width	0.004	0.801∗∗	0.671∗	0.815∗∗	−0.007	0.007	0.437	0.459	0.877∗∗	1

∗∗ P < 0.01.

∗ P < 0.05 level.

### 3.7 Principal component analysis of grain and nutritional traits

**Fig. 2:**
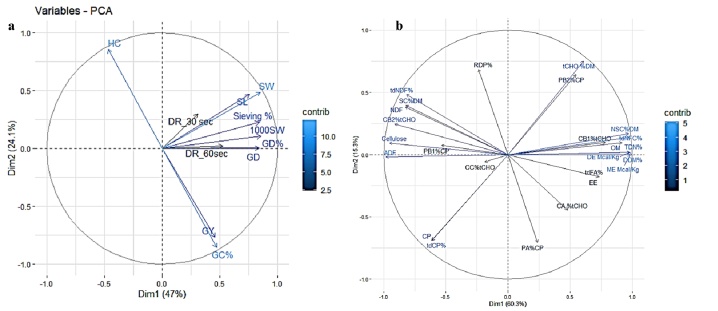
Principal component analysis (PCA) among eight oat genotypes: (a) grain yield and physical traits (b) chemical constituents and nutritional.

The principal component analysis (PCA) among oat varieties for the grain yield and physical traits accounted for 71.1 % of total variation. The PC1 explained 47 % of the total variance, with yield and milling traits as main contributors, while PC2, explained 24.1 % of the total variation with all measured traits being positively loaded. PCA showed that grain yield and groat content had the maximum contribution towards variation in the genotypes followed by sieving percentage, grain density, 1000 grain weight and seed length (Fig. 2a). Similarly, PCA among nutritional traits comprising energy, digestible nutrients, protein and carbohydrate fractions accounted for 75.6 % of total variation, where PC1 and PC2 explained 60.3 % and 15.3 % of the total variance, respectively (Fig. 2b).

## 4 Discussion

This study systematically characterized the oat varieties for grain yield, quality and digestibility related traits and revealed extensive genetic variation among oat varieties for the measured traits. The study identified genotypes with contrasting traits like JHO-2009-1 (high grain yield, high ME, TDN high, protein high, low ADF and high lignin) and JHO-851 (low grain yield, low lignin, low ME, TDN low, protein low and high ADF). These genotypes are valuable genetic resource for oat breeders to cross and select for superior genotypes and further investigate the genetics of grain yield and quality traits.

### 4.1 Yield, physical and milling traits of grain

Grain yield and groat percentage (2.15–5.35 t/ha and 64.77–69.2 %) of 25 oat genotypes [26] was higher, while thousand-grain weight (21.8–34.2) was lower than the observed values in the current study. Grain density (50.6–54.1 kg/hl) and thousand-grain weight (36.0–44.6 g) of ten oat cultivars [27] were relatively higher than values in present study, while hull contents (20.9–25.8) were lower (30.97–35.22 %). Nemeth et al. [28] reported (P < 0.01) significant difference in groat and hull% of oat grains (45.6–64.7 and 35.3–54.4 %). Howarth et al. [29] reported higher grain yield (6.03–10.49 t/ha), groat content (68.65–75.88 %), 1000 grain weight (35.52–48.18g) of oat varieties across 22 environments. Such differences across studies can be attributed to the nature of cultivars and screening environments. Grain yield (1.5–3.6 t/ha), groat content (74.2–79.3 %) and thousand-grain weight (30.2–33.3g) of ten oat cultivars [30] was similar to our results.

### 4.2 Chemical composition

Grain of oat varieties had CP (10.34–15.93 %), which is a favourable range as >7 % is required for rumen microbial growth and >15 % is needed for growth and milk production in cows [25]. Sterna et al. [31] reported CP and EE as 10.4–17.3 % and 8.05–8.82 %, respectively, in oat grains grown under different nitrogen levels. The EE (4.1–8.3 %) in our study is similar to the levels of 5.8–7.8 % in other oat varieties [32]. Similarly, results of our study agree with previous findings where CP (14.1–16.6 %) and EE (6.1–6.9 %), were reported in oat grains [33]. The grain mean CP and NDF of 12.71 and 30.0 %, respectively, of the varieties in our study are close to those of the National Research Council (NRC) [25]. The OM, ash, EE and CP in oat grains (96.62–97.09 %, 20.91–3.08 %, 4.16–6.66 % and 13.82–15.78 %) reported by Tosta [34] substantiates our results. Furthermore, NDF (21.74–23.43 %) is lower (28.29–31.58), while ADF and lignin (11.64–13.19 % and 1.55–3.27 %) agree with our findings [26]. Ash, CP and EE in grains of five oat cultivars in previous study (1.92–2.27 %, 9.94–10.9 % and 5.28–6.3 %; [35]) were lower, while NDF, ADF, cellulose and lignin (29.7–38.03 %, 14.1–18.37 %, 11.35–15.01 % and 2.72–3.6 %) were higher than values in this study. Oat grains CP, EE, NDF, ADF and cellulose (9.82, 4.83, 24.93, 8.51 and 5.27 %; [36]) were lower than values in this study. Lignin percent and NDF in grains of evaluated oat varieties lies within 7.11–14.55 % [34]. Lignin percent and NDF in grains is less than Sniffin et al. [17] for oat grain (9.4 %) except JHO99-1 (11.06 %) and this might be due to different genotypic constitution of cultivars in the studies. The NDIN (1.94 % DM) and ADIN (0.33 % DM) in oat grains in previous study [25] was higher than mean NDIN and ADIN values (0.34 % and 0.07 % DM) in this study. In contrast, NDIN and ADIN in oat grains (1.37 % and 0.26 % DM [33]) were lower than present values. The NDIP in grains of three oat cultivars (7.06–8.24 % CP [34]) was lower, while ADIP (1.69–3.25 % CP) was similar to values in present study. Hence, lack of agreement for some of the traits in the present study with previous studies can be attributed to use of different genotypes across studies.

### 4.3 Carbohydrate and protein fractions

In cereal grains, carbohydrates usually account for >70 % DM. Oat grains tCHO and NSC (76.92–78.22 % DM and 49.46–52.44 % DM) reported by Tosta [34] agree with our findings, however tCHO for oat grains (67.8 % DM; [37]) was lower in the present study. In contrast, Niu et al. [38] recorded relatively higher tCHO (77.7–81.75 % DM) in grains of three oat cultivars. Further, the C_A_, C_B1_, C_B2_ and C_C_ (5.39–16.65 %, 49.30–56.89 %, 19.80–33.35 %, and 6.53–11.67 % tCHO) of Niu et al. [28] substantiates the carbohydrate fraction values of our study. Carbohydrate fractions C_A_, C_B1_, C_B2_ + C_B3_ and C_C_ in grains of three oat cultivars (2.11–2.49 %, 61.73–64.87 %, 22.14–30.08 % and 5.12–10.52 % tCHO [34]) agree with our findings. The NSC (44.2–50.5 % DM in oat grains [33]) confirms with NSC values in our study.

Protein fractions P_A_, P_B1_ and P_B2_ in oat grains (59.06–62.20 %, 29.56–33.87 % and 4.70–5.90 % CP; [34]) were inconsistent, while P_C_ (1.69–3.25 % CP) was similar to our values. Protein fraction P_A_ (7.3–15.0 % CP) in oat grains substantiates our P_A_ values. Protein fractions P_A_ and P_C_ for oat grains (9.88–15.62 % and 4.18–5.07 % CP; [38]) agree with our findings (7.76–19.90 % and 2.19–4.08 % CP), while P_B1_, P_B2_ and P_B3_ values does not agree with our results. Such inconsistencies across studies are due to the genotype specific behaviour and nature of testing environments.

### 4.4 Energy and digestible nutrients

Oat grains GE (4.65–4.71 Mcal/kg DM [33]; is consistent with our GE values. TDN, DE and NE_L_ (92.62 %, 4.08 and 2.15 Mcal/kg DM) in the present study is more than that reported by the NRC [25]. The study identified higher oat grain NE_M_ and NE_G_ as compared to previous study [39] (2.03 and 1.37 Mcal/kg DM, respectively). Das et al. [36] reported lower TDN, DE and ME in oat grains (80.72, 3.50 and 3.14). The DE, ME, NE_M_, NE_L_ and NE_G_ in oat grains (3.69–3.91, 3.03–3.21, 2.05–2.20, 1.91–2.08 and 1.39–1.52 Mcal/kg DM [34]) tended to be marginally lower. The TDN in grains of oat varieties (82.92–88.88 % and 85.0–86.5 %) reported earlier [33,34] were 2–4 units lower than our TDN values. The higher TDN and energy in grains of our oat varieties may be attributed to their higher EE and lower ADF and lignin. Herrera-Saldana et al. [40] reported 80 % and >90 % oat grains *in sacco* DM and CP degradability, respectively within 2 h of incubation. Higher DDM for grains of JHO-822 and JHO2009–1 may be attributed to lower NDF, ADF, cellulose and lignin, while lowest DDM for JHO99–2 may be ascribed to its high fiber content. The tdCP, tdFA and tdNDF in oat grains (9.67, 3.83 and 11.22 % [36]) were lower, while tdNFC were higher than our values (44.87 %). Tosta [34] reported tdCP, tdFA, tdNDF and tdNFC (13.69–15.58, 3.16–5.66, 10.38–12.55 and 55.89–57.59 % DM) in grains of three oat varieties. Oat grains tdCP and tdFA (13.6–15.2 and 4.4–5.5 %; [33]) were similar, while tdNDF and tdNFC were higher and lower, respectively than values in present study.

### 4.5 Correlation of chemical constituents with energy and digestible nutrients

Concentrates ash, CP, NDF and lignin show a negative association with TDN, while EE and NFC is positively associated with TDN [41] which is consistent with correlation results in the present study. Like our study, CP of barley grains showed a negative correlation with DOM, DE and ME [42]. Lanzas et al. [43] reported that EE of cereal grains is strongly related to ME supply. The NDF of a fraction of NSC decreases diet energy as in barley grain NDF (r = −0.62) for predicting ME supply. Information on the correlation of carbohydrate and protein fractions of cereal grains with energy and degradable nutrients is scanty, however Serrapica et al. [44] reported the correlation of C_A_, C_B1_, C_B2_ and C_C_ carbohydrate and P_A_, P_B1_, P_B2_, P_B3_, P_C_ protein fractions with RDP, *in vitro* DM degradability and *in vitro* NDF degradability in oil seeds cakes which is inconsistent to our results, probably due to differences in carbohydrate and protein fractions concentration among feeds. A positive correlation of P_B1_ with RDP and tdCP is due to the fact that P_B1_ is rapidly degradable, which contributes to RDP and tdCP. A positive correlation (*p* < 0.01) of C_B1_ with TDN, DE, ME and tdNFC confirm that this fraction contains mainly starch (60 %) of NFC in cereal grains. In contrast, C_B2_ which mainly contains NDF is negatively correlated (*p* < 0.01) with TDN, DE, ME and tdNFC as NDF is a structural carbohydrate and shows negative effect on energy.

### 4.6 Correlation studies among grain yield and milling traits

Improvement in characters for grain yield and milling can be manifested based on these results which offer the possibility of simultaneous selection of these correlated traits for genetic enhancements in oats for yield and quality. A significant positive correlation between grain yield and groat percentage is crucial for selecting genotypes having high-quality standards and corroborates with results from previous studies in oat [45]. Grain density is also associated with groat percent, hull percent and grain compactness. These grain physical characteristics are correlated with 1000 grain weight, which is a highly heritable trait and remains in top priority in breeding programs [26]. Higher grain length (having longer grains) allows for more air spaces between kernels and eventually leads to lower test weights. Doehlert et al. [46] indicated a negative correlation between test weight and kernel length but failed to show a significant correlation between test weight and kernel width. The overall result of this study helps to identify the most relevant characters and their contributions and also visualizes the relationship among different traits evaluated for yield and milling quality.

In nutshell, JHO-2000-4, JHO-822 and JHO-2009-1 are superior oat varieties based on better grain yield, nutritional and digestibility associated traits. Interestingly, these three oat varieties clustered in a single group based on correlation of grain yield and physical traits. These oat varieties can be crossed and selection can be practiced to develop high grain yield and nutritionally rich oat varieties. Contrastingly, JHO-851 formed an outlier group and had low grain yield, low ME, TDN low, protein low, high ADF and low lignin. Hence JHO-851 can be crossed with JHO-2009-1 (high yield, high ME, TDN high, protein high, low ADF and high lignin) to develop mapping population and characterize the genomic regions for grain yield, quality and digestibility traits.

## 5 Conclusions

The current investigation for assessment of genetic variability in diverse oat varieties reveals significant genetic variation for grain yield and nutritional quality. The results provides a good opportunity in the field of oat breeding for the physical and nutritional traits. Grains had different contents of digestible dry matter, rumen degradable protein and truly digestible nutrients. Overall, this study provided valuable genetic resources and information on trait correlations, which has opened new potential avenues for oat breeding. Furthemore, the generated knowledge on oat varieties will be helpful to recommend better varieties for cultivation to farmers, cross and select for superior progenies and investigate the genetics of grain yield and quality traits. These insights further establish that oats, based on their physicochemical and nutritional traits, can be effectively utilized for livestock feed, food, and industrial applications.

## Additional information

No additional information is available for this paper.

## Data availability statement

Data will be made available on request.

## CRediT authorship contribution statement

**Sultan Singh:** Writing – review & editing, Writing – original draft, Supervision, Methodology, Investigation, Formal analysis, Data curation, Conceptualization. **Pushpendra Koli:** Writing – review & editing, Writing – original draft, Methodology, Formal analysis, Conceptualization. **Shahid Ahmed:** Supervision, Data curation, Conceptualization. **Neeraj Kumar:** Writing – original draft, Software, Conceptualization. **Maneet Rana:** Writing – review & editing, Writing – original draft, Conceptualization. **Rajesh Singhal:** Writing – original draft, Formal analysis. **Indu:** Writing – original draft, Software, Methodology, Writing – original draft, Software, Methodology. **Mukesh Choudhary:** Writing – review & editing, Writing – original draft, Investigation, Conceptualization. **Yonglin Ren:** Writing – review & editing, Writing – original draft, Supervision, Conceptualization.

## Declaration of competing interest

The authors declare that they have no known competing financial interests or personal relationships that could have appeared to influence the work reported in this paper.

## Abbreviations

ADF: acid detergent fiber
ADIN: Acid detergent insoluble nitrogen
C_A_: rapidly degradable sugars
C_B1_: intermediately degradable starch and pectins
C_B2_: slowly degradable cell wall
C_C_: unavailable/lignin-bound cell wall
CP: crude protein
DDM: digestible dry matter
DE: digestible energy
EE: ether extract
GE: gross energy
ME: metabolizable energy
NDF: neutral detergent fiber
NDIN: Neutral detergent insoluble nitrogen
NE_L_: net energy for lactation
NE_G_: net energy for growth/gain
NE_M_: net energy for maintenance
NSC: non-structural carbohydrates
OM: organic matter
P_A_: non-protein nitrogen
P_B1_: buffer soluble protein
P_B2_: neutral detergent soluble protein
P_B3_: acid detergent soluble protein
P_C_: indigestible/lignin bound protein
RDP: rumen degradable protein
SC: structural carbohydrates
SEM: standard error of the mean
tCHO: total carbohydrates
TDN: total digestible nutrients
tdCP: truly digestible crude protein
tdFA: truly digestible fat
tdNDF: truly digestible neutral detergent fiber
tdNFC: truly digestible non-fibrous carbohydrates
